# Short-lag spatial coherence imaging using minimum variance beamforming on dual apertures

**DOI:** 10.1186/s12938-019-0671-0

**Published:** 2019-04-23

**Authors:** Yanxing Qi, Yuanyuan Wang, Jinhua Yu, Yi Guo

**Affiliations:** 1 Department of Electronic Engineering, Fudan University, Shanghai, China; 2 Key Laboratory of Medical Imaging Computing and Computer Assisted Intervention (MICCAI) of Shanghai, Shanghai, China

**Keywords:** Short-lag spatial coherence, Synthetic aperture, Minimum variance, Adaptive beamforming, Ultrasound imaging

## Abstract

**Background:**

Short-lag spatial coherence (SLSC) imaging, a newly proposed ultrasound imaging scheme, can offer a higher lesion detectability than conventional B-mode imaging. It requires a high focusing quality which can be satisfied by the synthetic aperture imaging mode. However, traditional nonadaptive synthesis for the SLSC still offers an unsatisfactory resolution. The spatial coherence estimation on the receive aperture cannot fully utilize the coherence information in two-dimensional (2D) echo data.

**Methods:**

To overcome these drawbacks, an improved SLSC scheme with adaptive synthesis on dual apertures is proposed in this paper. The minimum variance (MV) beamformer is applied in synthesizing both the receiving and transmitting apertures, while the SLSC function is estimated on both apertures as well. In this way, the resolution is enhanced by the MV implementation, while the coherence in dual apertures is fully utilized.

**Results:**

Simulations, phantom experiments, and in vivo studies are conducted to evaluate the performance of the proposed method. Results demonstrate that the proposed method achieves the best performance in terms of the contrast ratio (CR), contrast-to-noise ratio (CNR), and the speckle signal-to-noise ratio (SNR). Specifically, compared with the delay-and-sum (DAS) method, the proposed method achieves 42.5% higher CR, 412.7% higher CNR, and 402.9% higher speckle SNR in simulations. The resolution is also better than the DAS and conventional SLSC beamformers.

**Conclusions:**

The proposed method is a promising technique for improving the SLSC imaging quality and can provide better visualization for medical diagnosis.

## Introduction

Short-lag spatial coherence (SLSC) imaging is a newly proposed beamforming technique for medical ultrasound imaging [1, 2]. Different from traditional beamforming methods, it compounds the spatial coherence rather than the magnitude of the array signal. Compared with conventional B-mode methods, the SLSC can better detect lesions inside tissues [1]. In addition, it can provide satisfactory contrast and lesion detectability even under a low signal-to-noise (SNR) condition [3]. Previous research has proven that the SLSC imaging can be applied to clinical research and has the potential of obtaining better imaging quality [4–6].

The Van Cittert–Zernike theorem is the basis of the SLSC method [2, 7, 8]. It illustrates that the good performance of the SLSC can only be achieved when the transmitted wave is sufficiently focused. In consideration of this principle, a substantial number of conventional imaging modalities are not suitable for the SLSC application. For instance, the commonly used line scan mode can achieve sufficient focus around the focal depth. However, beams are highly diffracted in the near-field and the deep regions, which means that the image quality inside these areas could suffer a severe degradation [9]. This limits the diagnostic value of the SLSC imaging. As another example, the acoustic wave in the plane wave imaging (PWI) is divergent. Consequently, the combination of the PWI and the SLSC results in poor imaging quality. To meet the requirements of the high focusing quality, it was suggested to apply the synthetic aperture (SA) focusing for the SLSC imaging [9]. Since multitransmission imaging modalities such as the SA imaging and the plane wave compounding (PWC) can naturally achieve the high focusing quality by the dynamic aperture focusing, the application of the SA imaging could improve the SLSC imaging quality mainly in terms of higher contrast [9].

Nevertheless, the inherent drawback of the SLSC imaging still persists. Despite the application of multitransmission modalities, the SLSC images still suffer a lower resolution than conventional B-mode ones [3]. To improve the resolution, one way is to modify the physical setup of the SLSC, such as changing the array aperture. Another alternative is to select an appropriate lag value, which is a vital parameter in the SLSC beamforming. Previous research confirmed that a large lag value could bring a comparably better resolution [2]. However, a small lag value is commonly adopted for a higher contrast-to-noise ratio (CNR) to distinguish hyperechoic structures. In addition, the small lag value is also necessary for a high SNR [1, 3]. How to make a compromise between the resolution and the CNR is still a conundrum. To pursue a better SLSC imaging quality, several groups including ours proposed some improved algorithms from another perspective [10, 11]. Zhao et al. [11] proposed a novel method which adaptively synthesizes the transmit (Tx) aperture using a minimum variance (MV) beamformer and then calculates the spatial coherence function. According to previous simulations and experiments, the integration of MV and SLSC could bring better resolution and speckle performance, which give us inspiration for further study.

To improve the resolution of the SLSC, we propose an improved SLSC imaging method using adaptive MV beamforming on dual apertures (DA-MV SLSC). In this method, the echo signals of the SA imaging are first recorded and written into a two-dimensional (2D) echo data matrix. Then the MV method is applied in beamforming the Tx aperture, and the spatial coherence is estimated in the receive (Rx) aperture. Inspired by the joint-transmitting-receiving beamformer [12], we implement another MV SLSC process but this time, the Rx aperture is MV beamformed, while the SLSC calculation is through the Tx aperture. Finally, the short-lag coherences of both apertures are summed to generate the SLSC image. The innovation point of the method comes from two aspects. First, the MV weighting process is integrated with the SLSC imaging to improve the focusing quality. Although the combination of the MV and the coherence factor (CF) can be frequently found in previous research, the proposed method is conceptually different. Unlike the conventional B-mode MV beamformers with a CF-based postfilter, it is still an SLSC imaging scheme which adopts the MV algorithm for adaptive aperture synthesizing. Second, the spatial coherences are calculated in both the Tx and Rx apertures, which fully utilize the coherence information contained in the 2D data matrix. To evaluate the proposed method, simulations, experiments, and in vivo studies are conducted. Results demonstrate that the proposed method performs better in the resolution and contrast. Detailed analysis will follow in the sections on results and discussion.

The rest part of this paper is organized as follows: “Backgrounds” section presents the background of some previous methods. “Methods” section describes the algorithm of the proposed method and the experimental setup. In “Results” section, the simulated and experimental studies are illustrated in detail, including the comparison between different methods. Finally, in “Discussion” section, the improvements of the newly proposed method versus the performance of the conventional B-mode imaging method, as well as the advantages of the former, are discussed, and the conclusions drawn based on the findings of this study with the suggested scope for future study are given in “Conclusion” section.

## Backgrounds

### Synthetic aperture imaging model

The SA imaging originates from the radar system and is then applied into medical ultrasound imaging [13–15]. In this paper, the synthetic transmit aperture technique is used, which means that only one element fires for each transmitting event. After the propagation through the region of interest (ROI), the backscattered echoes are recorded by all elements. The firing events switch through all transmitting elements to synthesize the full aperture. Suppose an *M*-element array is adopted, we can acquire an $M \times M$ size 2D echo data matrix after all data acquisitions:

1 $$\varvec{X}\left( n \right) = \left[ {\begin{array}{*{20}ll} {x_{{1,1}} \left( n \right)} & {x_{{1,2}} \left( n \right)} & \cdots & {x_{{1,M}} \left( n \right)} \\ {x_{{2,1}} \left( n \right)} & {x_{{2,2}} \left( n \right)} & \ldots & {x_{{2,M}} \left( n \right)} \\ { \vdots ~} & \vdots & \ddots & \vdots \\ {x_{{M,1}} \left( n \right)} & {x_{{M,1}} \left( n \right)} & \cdots & {x_{{M,M}} \left( n \right)} \\ \end{array} } \right] ,$$

where *n* is the time step and $x_{i,j} \left( n \right)$ represents the echo data recorded by the *j*th element when using the *i*th transmitting element. An appropriate time delay is compensated into each channel. In the traditional delay-and-sum (DAS) algorithm, for each imaging point, all channel data are averaged through rows to generate low-resolution images at first. Then these images can be averaged for the final high-resolution image:

2 $$\begin{array}{*{20}l} {z_{\text{DAS}} = \frac{1}{M}\mathop \sum \limits_{i = 1}^{M} \omega_{\text{T}} \left( i \right)z_{\text{LRI}}^{i} = \frac{1}{{M^{2} }}\mathop \sum \limits_{i = 1}^{M} \omega_{\text{T}} \left( i \right)\mathop \sum \limits_{j = 1}^{M} \omega_{\text{R}} \left( j \right)x_{j}^{i} \left( {n_{i,j} } \right),} \\ \end{array}$$

where $\omega_{\text{R}}$ is the receiving apodization window and $\omega_{\text{T}}$ is the transmitting one. The $n_{i,j}$ stands for the time index of $x_{i,j} \left( n \right)$, which is calculated by the positions of the transmitting element, the receiving element, and the imaging point.

### Short-lag spatial coherence algorithm

For echo signals of one single emission, the SLSC imaging algorithm differs a lot from the DAS beamformer. When a transducer array with *M* elements is adopted, the backscattered echo arriving at the array after a firing event could be expressed as

3 $$\begin{array}{*{20}c} {\varvec{x}\left( n \right) = \left[ {x_{1} \left( n \right),x_{2} \left( n \right), \ldots ,x_{M} \left( n \right)} \right]^{\text{T}} } \\ \end{array},$$

where $x_{j} \left( n \right)$ represents the echo signal received by the *j*th element with an appropriated time delay for each channel, *n* is the time index, and $\left( \cdot \right)^{\text{T}}$ stands for the matrix transpose. Then the mutual intensity or the so-called spatial coherence between two receiving elements could be estimated from their cross-correlations:

4 $$\begin{array}{*{20}c} {\hat{R}\left( m \right) = \frac{1}{M - m}\mathop \sum \limits_{i = 1}^{M - m} \frac{{\mathop \sum \nolimits_{{n = n_{1} }}^{{n_{2} }} x_{i} \left( n \right)x_{i + m} \left( n \right)}}{{\sqrt {\mathop \sum \nolimits_{{n = n_{1} }}^{{n_{2} }} x_{i}^{2} \left( n \right)\mathop \sum \nolimits_{{n = n_{1} }}^{{n_{2} }} x_{i + m}^{2} \left( n \right)} }}} \\ \end{array},$$

where *m* is the distance or lag between two elements in terms of number of elements. The temporal sampling kernel $n_{2} - n_{1}$ is usually shorter than one cycle [2, 3, 9]. The final SLSC value is calculated using the integral of the first *q* lags:

5 $$\begin{array}{*{20}c} {R_{\text{SLSC}} = \mathop \sum \limits_{m = 1}^{q} \hat{R}\left( m \right)} \\ \end{array},$$

where *q* is the number of lags used for summation. An important parameter for the SLSC is *Q *= *q*/*M *× 100%, which is defined as the ratio between the lag number and the element number. Generally speaking, the lower the *Q* is, the less the high-frequency component is taken into account, which means the contrast would increase, while the resolution could be damaged, and vice versa [2]. Normally the parameter *Q* is set to range from 10% to 30%, in order to make a compromise between the resolution and contrast [2, 3].

The SLSC is based on the assumption that the focusing errors due to the acoustic clutter mainly influence the correlations of the “short-lag” elements which are closely separated [2]. This assumption illustrates why only signals of closed elements are used to compute the spatial coherence function. Another premise of the SLSC is that the hyperechoic target and the speckle region have a great difference in their spatial coherence function, which means that a high focusing quality is a prerequisite [2]. The conventional line scan ultrasound imaging mode could only ensure the satisfactory focusing quality near the focal zone, while the short-lag coherence function severely decreases in the region far away from the focus [9]. Hence, the SLSC imaging quality suffers a great degradation in these regions. As a solution, the SA imaging modality can help handle this drawback since the SA imaging dataset could be dynamically focused for each imaging point. In addition, on the basis of the acoustic reciprocity, the spatial coherence can be calculated in either the synthetic Tx aperture or the Rx aperture [16], which is this paper’s starting point.

### Minimum variance beamformer

Adaptive beamformers replace the uniform weight used in the DAS beamformer with a data-dependent weighting vector $\varvec{w}\left( n \right)$ for the signal summation. The most representative one, MV beamformer, was originally proposed by Capon in 1969 [17]. It calculates the adaptive weights by minimizing the output energy subject to the constraint that the desired signal is distortionless. Under the incoherent premise between the signal and noise, it is theoretically equal to minimizing the output noise. The MV beamformed output is a product of the received signal vector and the weighting vector:

6 $$\begin{array}{*{20}c} {z\left( n \right) = \varvec{w}^{\text{H}} \left( n \right)x\left( n \right),} \\ \end{array}$$

where $\varvec{x}\left( n \right)$ is the echo signal in Eq. (3), $\varvec{w}\left( n \right)$ is the MV weighting vector, and $\left( \cdot \right)^{\text{H}}$ stands for the conjugate transpose. Then the MV beamforming process could be expressed by

7 $$\begin{array}{*{20}c} {\mathop {\hbox{min} }\limits_{{\mathbf{w}}} \varvec{w}^{\text{H}} \varvec{R}\left( n \right)\varvec{w}, \;{\text{subject to}} \;\varvec{w}^{\text{H}} a = 1, } \\ \end{array}$$

where $\varvec{a}$ stands for the steering vector, which can all be assumed as one vector after we apply the time delay to each channel. $\varvec{R}\left( n \right)$ is the covariance matrix of the array data. Using the Lagrange method, the solution to (7) can be calculated as below:

8 $$\begin{array}{*{20}c} {\varvec{w}_{\text{MV}} = \frac{{\varvec{R}^{ - 1} \varvec{a}}}{{\varvec{a}^{\text{H}} \varvec{R}^{ - 1} \varvec{a}}}.} \\ \end{array}$$

In practice, the covariance matrix $\varvec{R}\left( n \right)$ is usually unknown and should be estimated by the temporal array samples. A simple approximation could be $\varvec{R}\left( n \right) = E\left[ {\varvec{x}\left( n \right)\varvec{x}^{\text{H}} \left( n \right)} \right]$ on the basis of the assumption that the signal and noise are uncorrelated. However, in practical ultrasound imaging, the signal and noise are usually highly correlated. To enhance the robustness, the spatial smoothing is proposed [18, 19]. It divides the array into several overlapped short subarrays and averages the covariance matrix through all subarrays. In this way, the on-axis signals can be decorrelated from off-axis ones. The spatial smoothing could be expressed as

9 $$\begin{array}{*{20}c} {\varvec{R}\left( n \right) = \frac{1}{M - L + 1}\mathop \sum \limits_{l = 1}^{M - L + 1} \varvec{x}_{l} \left( n \right)\varvec{x}_{l}^{\text{H}} \left( n \right),} \\ \end{array}$$

where $\varvec{x}_{l} = \left[ {x_{l} \left( n \right),x_{l + 1} \left( n \right), \ldots ,x_{l + L - 1} \left( n \right)} \right]^{\text{T}}$ stands for the *l*th subarray. *L* is the subarray length and is usually a little smaller than half of the array size [19, 20]. Another frequently used method to improve the robustness is the diagonal loading. It adds a small constant to the original covariance matrix: $\varvec{R}_{\text{DL}} \left( n \right) = \varvec{R}\left( n \right) + \varepsilon \cdot \varvec{I}$, which can make the covariance matrix nonsingular. Here $\varepsilon$ is a small constant, and $\varvec{I}$ is the identity matrix. The constant $\varepsilon$ is often set to be $\Delta \cdot {\text{trace}}\left( {\varvec{R}\left( n \right)} \right)$, while $\Delta$ is usually less than 0.1 [19, 20].

Using the modified covariance matrix $\varvec{R}_{\text{DL}} \left( n \right)$, the MV weighting vector $\varvec{w}_{\text{MV}}$ can be computed using Eq. (8). Considering the spatial smoothing technique, Eq. (6) should be rewritten to form

10 $$\begin{array}{*{20}c} {z_{\text{MV}} \left( n \right) = \frac{1}{M - L + 1}\mathop \sum \limits_{l = 1}^{M - L + 1} \varvec{w}_{\text{MV}}^{\text{H}} \left( n \right)\varvec{x}_{l} \left( n \right)} \\ \end{array}$$

## Methods

### Transmit aperture weighting for the SLSC imaging

The adaptive beamformer can be integrated with the SLSC process. Accordingly, a two-step signal-processing procedure consisting of both the MV process and the SLSC imaging is logically proposed. The SA dataset in “Synthetic aperture imaging model” section, with the appropriate time delay compensation, can be obtained after data collection. The first step is to conduct the adaptive focusing process in the Tx aperture using the MV beamformer. After synthesizing the Tx aperture, the second step is to calculate the spatial coherence in the Rx aperture [11].

In the first step, the 2D echo data matrix is MV beamformed in the Tx dimension to synthesize the Rx aperture. To calculate the Tx–MV weights, the covariance matrix of the Tx aperture is averaged through all Rx array elements on the basis of the multiwave beamforming approach [21, 22]. In addition, since a temporal kernel is adopted in the SLSC algorithm, it is also necessary to adopt the temporal smoothing in the MV process, which averages the covariance matrix through the time index $n_{1}$ to $n_{2}$ (the temporal kernel). After all estimations, the covariance matrix should become as

11 $$\begin{array}{*{20}c} {\varvec{R}_{\text{Tx}} \left( n \right) = \frac{1}{{\left( {n_{2} - n_{1} } \right)M\left( {M - L + 1} \right)}}\mathop \sum \limits_{{n = n_{1} }}^{{n_{2} }} \mathop \sum \limits_{j = 1}^{M} \mathop \sum \limits_{l = 1}^{M - L + 1} \varvec{x}_{{{\text{Tx}}_{j} ,l}} \left( n \right)\varvec{x}_{{{\text{Tx}}_{j} ,l}}^{\text{H}} \left( n \right),} \\ \end{array}$$

where $\varvec{x}_{{{\text{Tx}}_{j} ,l}} \left( n \right) = \left[ {x_{l,j} \left( n \right),x_{l + 1,j} \left( n \right), \ldots ,x_{l + L - 1,j} \left( n \right)} \right]^{\text{T}}$ represents the *l*th Tx subarray of the *j*th Rx element data. Previous research has proven that the averaging process on both Tx aperture and Rx elements could help increase the accuracy of the estimation, which improves the imaging quality [12, 22]. Then the Tx–MV weight $\varvec{w}_{{{\text{Tx}} - {\text{MV}}}} \left( n \right)$ can be calculated by Eq. (8) using the estimated $\varvec{R}_{\text{Tx}} \left( n \right)$ with the appropriate diagonal loading. After the MV weighting process in the Tx aperture, the Tx-synthesized Rx aperture can be written as $\varvec{z}_{\text{Rx}} \left( n \right) = \left[ {z_{{{\text{Rx}}_{1} }} \left( n \right),z_{{{\text{Rx}}_{2} }} \left( n \right), \ldots ,z_{{{\text{Rx}}_{M} }} \left( n \right)} \right]$, where the Tx-weighted output of a single Rx channel $z_{{{\text{Rx}}_{j} }} \left( n \right)$ can be calculated as

12 $$\begin{array}{*{20}c} {z_{{{\text{Rx}}_{j} }} \left( n \right) = \frac{1}{{\left( {n_{2} - n_{1} } \right)\left( {M - L + 1} \right)}}\mathop \sum \limits_{{n = n_{1} }}^{{n_{2} }} \mathop \sum \limits_{l = 1}^{M - L + 1} \varvec{w}_{{{\text{Tx}} - {\text{MV}}}}^{\text{H}} \left( {{n}} \right)\varvec{x}_{{{\text{Tx}}_{j} ,l}} \left( n \right)} \\ \end{array}.$$

The second step is to estimate the spatial coherence through the Rx aperture, namely, the Rx spatial coherence function, $\hat{R}_{\text{Rx}} \left( m \right)$:

13 $$\begin{array}{*{20}c} {\hat{R}_{\text{Rx}} \left( m \right) = \frac{1}{M - m}\mathop \sum \limits_{i = 1}^{M - m} \frac{{\mathop \sum \nolimits_{{n = n_{1} }}^{{n_{2} }} z_{{{\text{Rx}}_{i} }} \left( n \right)z_{{{\text{Rx}}_{i + m} }} \left( n \right)}}{{\sqrt {\mathop \sum \nolimits_{{n = n_{1} }}^{{n_{2} }} z_{{{\text{Rx}}_{i} }}^{2} \left( n \right)\mathop \sum \nolimits_{{n = n_{1} }}^{{n_{2} }} z_{{{\text{Rx}}_{i + m} }}^{2} \left( n \right)} }}} \\ \end{array}.$$

### Receive aperture weighting for the SLSC imaging

On the basis of the acoustic reciprocity, the adaptive weighting process can be implemented in both the Tx aperture and the Rx one [16]. In “Transmit aperture weighting for the SLSC imaging” section, the adaptive weighting process is done in the Tx aperture, and the spatial coherence function is calculated through the Rx aperture. Correspondingly, another MV–SLSC process can be implemented. However, this time the Rx aperture focusing process is first conducted using the MV beamformer, and then the SLSC function could be estimated through the Tx aperture. Similarly, the covariance matrix of the Rx aperture is estimated by averaging through not only all Rx subarrays, but also all Tx array elements:

14 $$\begin{array}{*{20}c} {\varvec{R}_{\text{Rx}} \left( n \right) = \frac{1}{{\left( {n_{2} - n_{1} } \right)M\left( {M - L + 1} \right)}}\mathop \sum \limits_{{n = n_{1} }}^{{n_{2} }} \mathop \sum \limits_{i = 1}^{M} \mathop \sum \limits_{l = 1}^{M - L + 1} \varvec{x}_{{{\text{Rx}}_{i} ,l}} \left( n \right)\varvec{x}_{{{\text{Rx}}_{i} ,l}}^{\text{H}} \left( n \right),} \\ \end{array}$$

where $\varvec{x}_{{{\text{Rx}}_{i} ,l}} \left( n \right) = \left[ {x_{i,l} \left( n \right),x_{i,l + 1} \left( n \right), \ldots ,x_{i,l + L - 1} \left( n \right)} \right]^{\text{T}}$ represents the *l*th Rx subarray of the *i*th Tx element data. The Rx–MV weight $\varvec{w}_{{{\text{Rx}} - {\text{MV}}}} \left( n \right)$ can also be calculated by Eq. (8). After the MV weighting process in the Rx aperture, the Rx-synthesized Tx aperture can be written into $\varvec{z}_{\text{Tx}} \left( n \right) = \left[ {z_{{{\text{Tx}}_{1} }} \left( n \right),z_{{{\text{Tx}}_{2} }} \left( n \right), \ldots ,z_{{{\text{Tx}}_{M} }} \left( n \right)} \right]$, where the Rx-weighted output of a single Tx element $z_{{{\text{Tx}}_{i} }} \left( n \right)$ can be calculated as

15 $$\begin{array}{*{20}c} {z_{{{\text{Tx}}_{i} }} \left( n \right) = \frac{1}{{\left( {n_{2} - n_{1} } \right)\left( {M - L + 1} \right)}}\mathop \sum \limits_{{n = n_{1} }}^{{n_{2} }} \mathop \sum \limits_{l = 1}^{M - L + 1} \varvec{w}_{{{\text{Rx}} - {\text{MV}}}}^{\text{H}} \left( {{n}} \right)\varvec{x}_{{{\text{Rx}}_{i} ,l}} \left( n \right)} \\ \end{array}.$$

As mentioned earlier, the spatial coherence can also be estimated through the Tx aperture. The Tx spatial coherence function $\hat{R}_{\text{Tx}} \left( m \right)$ can be calculated as follows:

16 $$\begin{array}{*{20}c} {\hat{R}_{\text{Tx}} \left( m \right) = \frac{1}{M - m}\mathop \sum \limits_{i = 1}^{M - m} \frac{{\mathop \sum \nolimits_{{n = n_{1} }}^{{n_{2} }} z_{{{\text{Tx}}_{i} }} \left( n \right)z_{{{\text{Tx}}_{i + m} }} \left( n \right)}}{{\sqrt {\mathop \sum \nolimits_{{n = n_{1} }}^{{n_{2} }} z_{{{\text{Tx}}_{i} }}^{2} \left( n \right)\mathop \sum \nolimits_{{n = n_{1} }}^{{n_{2} }} z_{{{\text{Tx}}_{i + m} }}^{2} \left( n \right)} }}} \\ \end{array}$$

### SLSC image formation

The beamforming processes in “Transmit aperture weighting for the SLSC imaging” and “Receive aperture weighting for the SLSC imaging” sections are quite similar, or “symmetric” in a more precise way. If we transpose the SA data matrix in (1), and then repeat the MV SLSC process described in “Transmit aperture weighting for the SLSC imaging” section on the transposed matrix, we can find that both the procedure and the result are the same as described in the “Receive aperture weighting for the SLSC imaging” section. It does tally with the acoustic reciprocity mentioned before. There is no precedence in the calculation of the Rx spatial coherence or the Tx one. Both implementations are based on the original echo data (1). Reasonably both Rx and Tx spatial coherence functions can be used for the SLSC image formation.

Ordinarily, the SLSC image formation process can be realized through Eq. (5). In this paper, we adopt a modified version to accord with the aperture control technique [11]. The *f*-number, defined as the ratio between the imaging depth and the effective aperture size, is commonly used to keep a consistent resolution through all depths in medical ultrasound imaging. In this paper’s SA implementation, since the MV weighting process is conducted in both apertures and so is the SLSC estimation process, the same f-number value is adopted in both the Tx and Rx apertures in order to make two aperture sizes identical. Another reason is to meet the requirements of the reciprocity of the acoustics. An equal size of the transmitting and receiving apertures can maintain the same weight of two apertures in the SLSC calculation, which helps average and reduce the focusing errors in the two apertures. Since the aperture size varies through different imaging depths, the SLSC summation process should be weighted to maintain similar signal magnitudes in the whole imaging region. The modified version of Eq. (5) could be expressed as

17 $$\begin{array}{*{20}c} {R_{\text{SLSC}} = \frac{1}{q}\mathop \sum \limits_{m = 1}^{q} \left( {\hat{R}_{\text{Rx}} \left( m \right) + \hat{R}_{\text{Tx}} \left( m \right)} \right)} \\ \end{array}.$$

Since *q* is correlated with the imaging depth, the averaging factor 1/*q* can help keep a consistent signal magnitude in different depths in the final SLSC image.

### Implementation summary of the algorithm

To make the proposed scheme clear, we give a brief summary of the procedures here.

1. Transform the received radio frequency (RF) data to the in-phase and quadrature (IQ) domains with the appropriate time delay for each channel to obtain the echo data matrix (1).
2. Adaptively synthesize the Tx aperture using the MV beamformer by Eqs. (11) and (12). Then estimate the SLSC function in the Rx aperture using Eq. (13).
3. Accordingly, synthesize the Rx aperture by Eqs. (14) and (15) and estimate the Tx SLSC function using Eq. (16).
4. Sum both the Rx and Tx spatial coherence function by Eq. (17) for the SLSC image formation.
5. Repeat the procedure (1) to (4) for each imaging point.

After all beamforming process, the SLSC image could be directly displayed without a dynamic compression, which is different from the conventional B-mode imaging.

### Experimental setup

Both the simulations and experiments were conducted to evaluate the performance of the proposed method. Simulated data were acquired using the Matlab simulation tool Field II [23, 24]. All phantom and in vivo data were generated from the Verasonics ultrasound platform (V1, Verasonics, Redmond, Washington).

In the Field II simulation, a 5-MHz, 128-element transducer array with a 0.3 mm pitch was adopted. The sampling rate was 40 MHz and a two-cycle sinusoid was used for the excitation pulse. To study the point spread function (PSF), four point targets at *z* = 30 mm, 35 mm, 40 mm, and 45 mm were first simulated. A 10 dB zero-mean white Gaussian noise (WGN) was added into each channel to simulate the background noise. In the second simulation, two 2.5-mm radius circular anechoic cysts centered at *z* = 37 mm and *z* = 52 mm were simulated. The thickness of the cyst through the *y*-axis was set to be 8 mm. The speckle region was generated using randomly distributed and random amplitude scatterers with a total amplitude of 10 per wavelength cubic ($\lambda^{3}$).

In the phantom and in vivo experiments, a 5-MHz, 128-element linear transducer array with a 0.3 mm pitch (L11-4v, Verasonics, Redmond, Washington, USA) was used. The sampling frequency was originally set to 20 MHz, and the recorded RF data were then resampled at 40 MHz to maintain the same time accuracy with the simulations. All phantom data were obtained using a CIRS calibration phantom (Model 040GSE, Computerized Imaging Reference Systems Inc., Norfolk, Virginia, USA). In vivo human carotid artery data were acquired from a 28-year-old male volunteer.

For the SA imaging modality, a spherical wave was transmitted by a single element and recorded by all elements for each emission. The f-number was set to 1.4 using a rectangular window for both Tx and Rx apertures. A subarray length of *L *= 0.3*M* was used for the spatial smoothing process in the MV algorithm. The diagonal loading parameter $\Delta$ was set to be 0.01. For the SLSC procedure, the ratio *Q *= *q*/*M* was set to be 15% and the temporal kernel was the same as one cycle [2, 3, 9]. These parameters are presented in Table 1.

**Table 1:** Transducer and processing parameters

Parameter	Value
Sound speed *c*	1540 m/s
Number of array elements *M*	128
Element pitch	0.3 mm
Center frequency $f_{0}$	5 MHz
Sampling frequency $f_{s}$	40 MHz
*F* number	1.4
Subarray length *L*	0.3*M*
Diagonal loading factor $\Delta$	0.01
SLSC sampling kernel $n_{2} - n_{1}$	1 cycle
SLSC ratio *Q*	15%

Different results using the DAS, the MV, the SLSC and the proposed DA-MV SLSC are shown together to make an exhaustive comparison. To quantify the performance of different methods, the full-width at half-maximum (FWHM, defined as − 6 dB beam width for the mainlobe) was adopted for point targets, while the contrast ratio (CR), the CNR, and the speckle SNR were measured for cyst targets. The CR, CNR, and speckle SNR can be calculated by

18 $$\begin{array}{*{20}c} {{\text{CR}} = 20\log_{10} (\mu_{\text{c}} /\mu_{\text{b}} ),} \\ \end{array}$$

19 $$\begin{array}{*{20}c} {{\text{CNR}} = \frac{{\left| {\mu_{\text{b}} - \mu_{\text{c}} } \right|}}{{\sqrt {\sigma_{\text{b}}^{2} + \sigma_{\text{c}}^{2} } }},} \\ \end{array}$$

20 $$\begin{array}{*{20}c} {{\text{SNR}} = \frac{{\mu_{\text{b}} }}{{\sigma_{\text{b}} }},} \\ \end{array}$$

where $\mu_{\text{b}}$ and $\mu_{\text{c}}$ are the mean signal magnitudes of the speckle and cyst regions, $\sigma_{\text{b}}$ and $\sigma_{\text{c}}$ are the standard deviations of the signal magnitude in the speckle and cyst regions, respectively [2].

## Results

### Simulated study

**Fig. 1:**
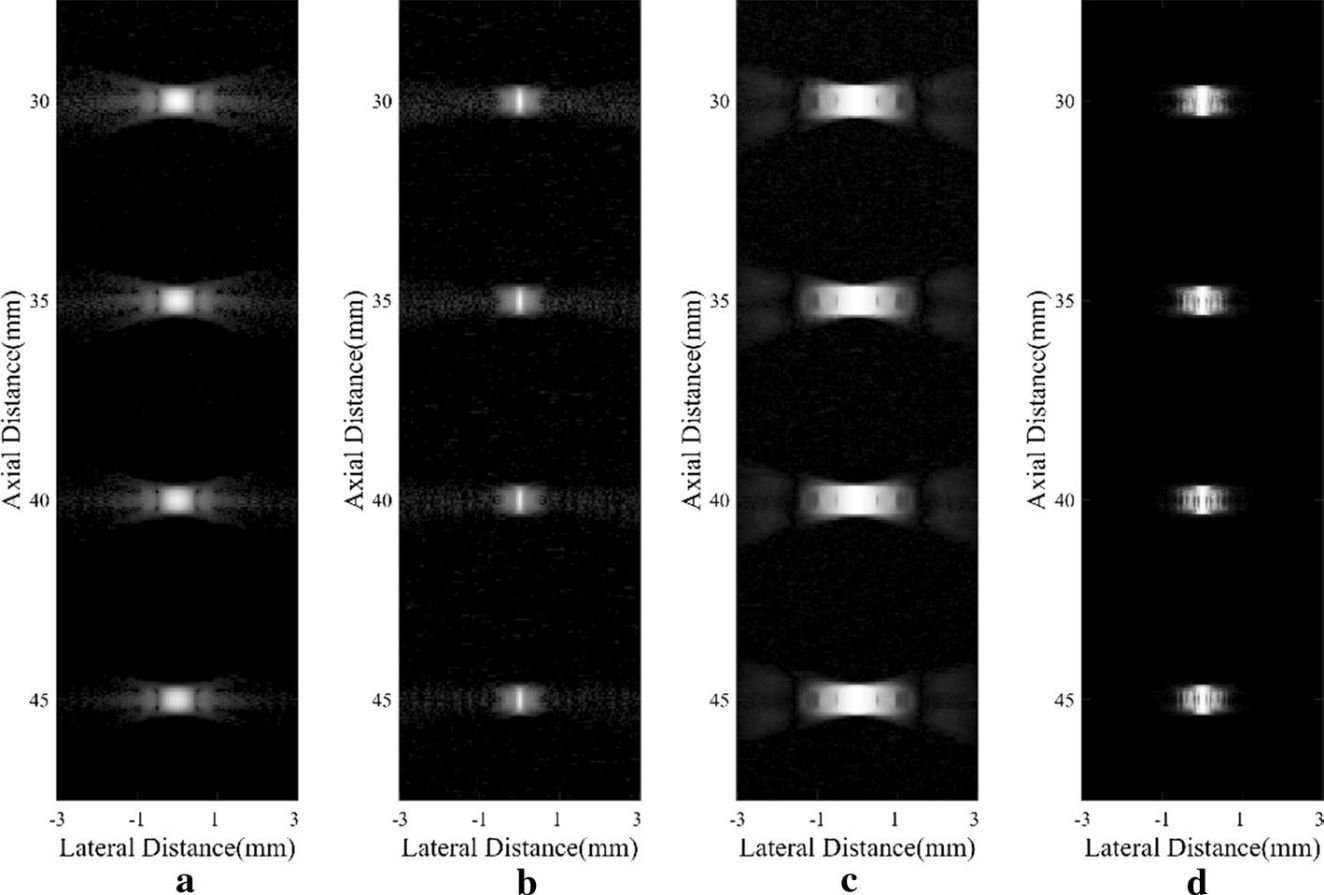
Simulated point scatterers: **a** DAS, **b** MV, **c** SLSC, **d** DA-MV SLSC. The **a** and **b** are B-mode images shown with a dynamic range of 60 dB

Figure 1 shows images of point targets using different methods. In Fig. 1a, sidelobes are visibly obvious with the DAS beamformer. Using the adaptive MV beamformer, sidelobes could be effectively suppressed in Fig. 1b. The original SLSC result in Fig. 1c seems to be worse than the DAS B-mode image since the points are wider. According to previous research, the wide mainlobe width could occur when the background noise level is very low [3]. As shown in Fig. 1d, the proposed DA-MV SLSC can narrow the mainlobe when compared with conventional SLSC method. The sidelobes are also suppressed to a low degree.

**Fig. 2:**
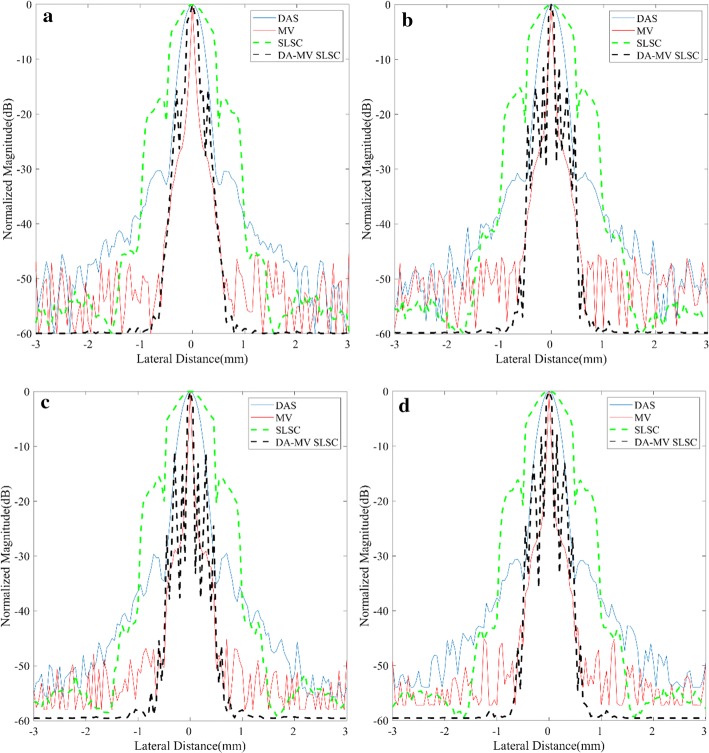
Lateral variations of different beamformed responses at **a** *z* = 30 mm, **b** *z* = 35 mm, **c** *z* = 40 mm, **d** *z* = 45 mm in the point target simulation

For quantitative measurements, plots of lateral variations of different methods at the depth *z* = 30, 35, 40 and 45 mm are shown in Fig. 2 while statistical results of corresponding FWHMs are presented in Table 2. It is shown that our method achieves better performance than not only the SLSC but also the DAS, which confirms the resolution advantage of the proposed method.

**Table 2:** Full-width at half-maximum (FWHM) for the beamformed responses at *z* = 30/35/40/45 mm

Beamformer	FWHM (mm)
DAS	0.42/0.41/0.42/0.41
MV	0.07/0.07/0.07/0.07
SLSC	0.88/0.85/0.90/0.87
DA-MV SLSC	0.13/0.12/0.13/0.12

**Fig. 3:**
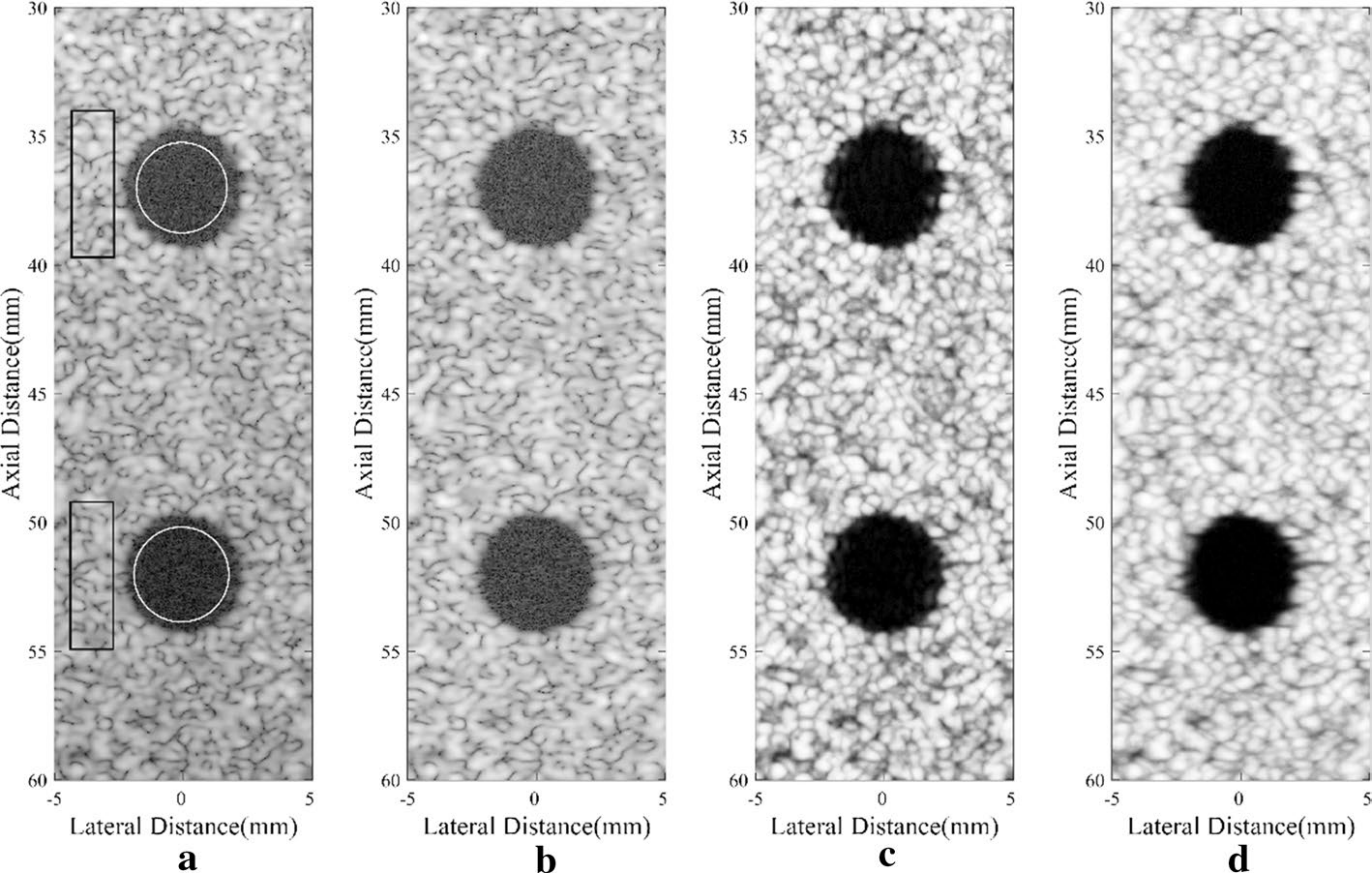
Simulated anechoic cysts: **a** DAS, **b** MV, **c** SLSC, **d** DA-MV SLSC. The **a** and **b** are B-mode images shown with a dynamic range of 60 dB

Figure 3 shows images of the simulated anechoic cysts by different beamformers. It could be observed that noises are still at a high level inside the cysts of Fig. 3a, which mainly comes from the sidelobes of the background speckle region. The MV beamformer can slightly suppress the noises while the SLSC can offer an effective noise suppression. In the comparison between Fig. 3c, d, both show a good visualization of the anechoic cysts while the boundaries of the cysts in Fig. 3d are more distinct than in Fig. 3c. This indicates the advantage of integrating the MV beamformer with the SLSC algorithm. In Fig. 3d, noises are highly suppressed inside cysts, and the speckle regions are smoother with less dark spots. Generally speaking, the proposed method obtains a satisfactory performance in the cyst simulation.

We measure the CR, CNR, and speckle SNR for both cysts, and the numerical results are shown in Table 3. The corresponding speckle region marked by a black rectangle is selected at the same depth of the cyst marked by a white circle to avoid different attenuation through depths [11]. As shown in the results, B-mode MV images do not show much improvement in three metrics. The SLSC method could bring the significant increment mainly in the CNR. In comparison, the DA-MV SLSC can increase three metrics and get a comparably better performance. Generally speaking, the noise inside cysts is remarkably suppressed by the proposed method, the CR has been notably increased. In addition, the speckle patterns are well preserved, which results in a higher speckle SNR.

**Table 3:** CR, CNR, and Speckle SNR of the simulated cysts centered at *z* = 37 mm/52 mm

Beamformer	CR (dB)	CNR	Speckle SNR
DAS	− 27.00/− 29.42	1.65/1.91	1.72/1.98
MV	− 26.65/− 28.68	1.69/1.90	1.78/1.97
SLSC	− 30.03/− 35.39	6.14/7.74	6.40/7.91
DA-MV SLSC	− 35.96/− 41.93	8.46/10.31	8.65/10.41

### Experimental phantoms

**Fig. 4:**
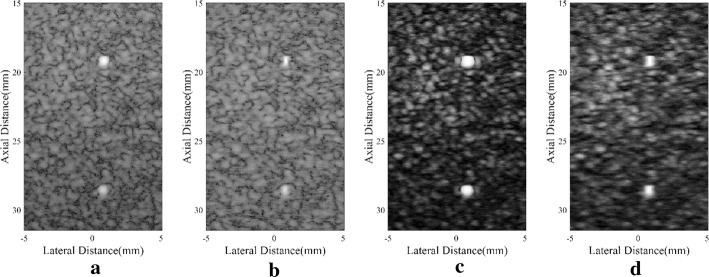
Experimental wire target phantom: **a** DAS, **b** MV, **c** SLSC, **d** DA-MV SLSC. The **a** and **b** are B-mode images shown with a dynamic range of 60 dB

Figure 4 shows images of experimental phantoms consisting of two wire targets. The experimental results are similar to the simulated ones in “Experimental phantoms” section. The MV beamformer can obtain a narrower mainlobe width than the DAS beamformer. The speckle performance of the MV beamformer is also visually better than that of the DAS. For the SLSC beamformer, hyperechoic point targets are visually more detectable, which can be viewed from Fig. 4c, d. In comparison with the original SLSC method, the proposed method obtains better performance mainly in the mainlobe width.

**Fig. 5:**
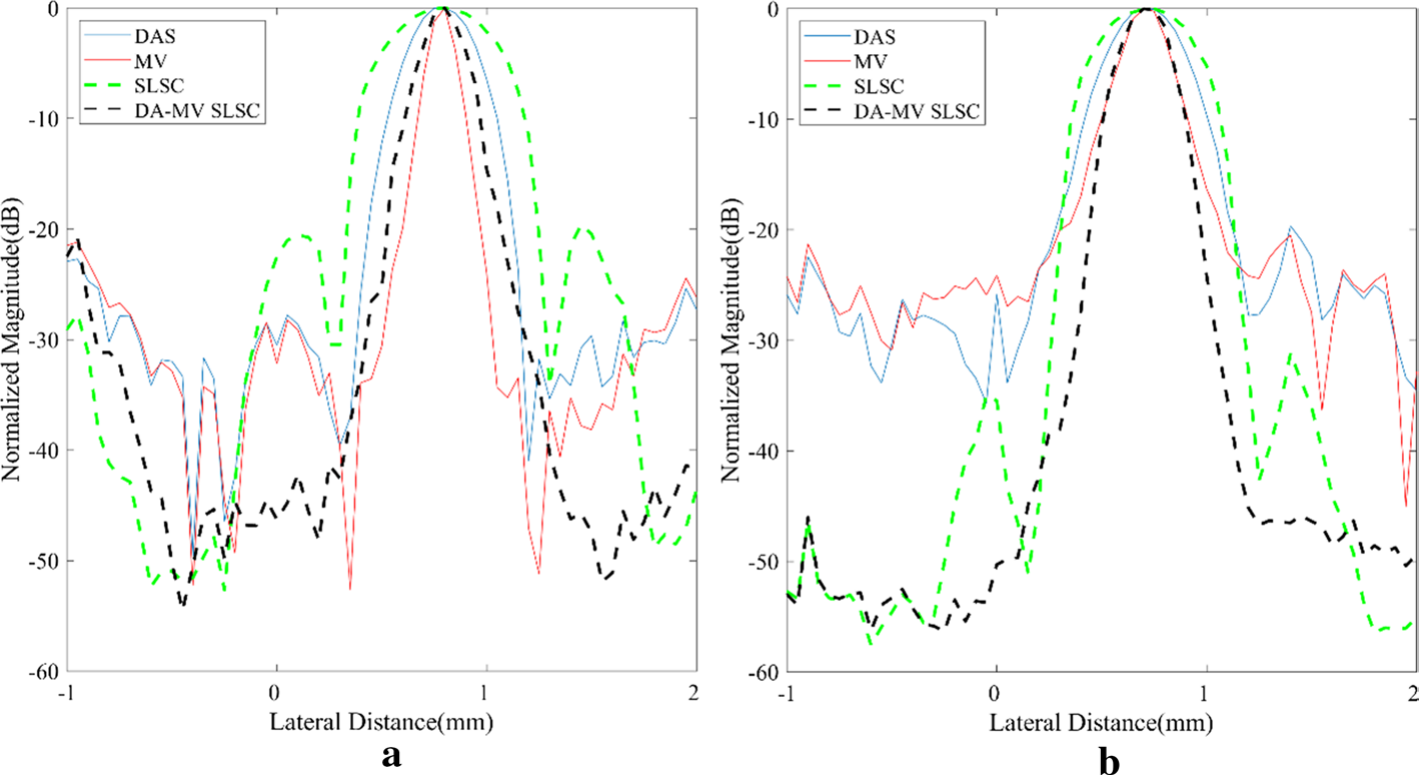
Lateral variations of different beamformed responses at **a** *z* = 19 mm, **b** *z* = 28 mm in the wire target phantom experiment

Figure 5 plots the lateral variations across the depth *z* = 19 mm and 28 mm, while statistical results are given in Table 4. The MV gets the narrowest FWHM among all methods. In the SLSC series, the proposed method achieves a good performance just as it does in the point target simulation.

**Table 4:** Full-width at half-maximum (FWHM) for the beamformed responses at *z* = 19/28 mm

Beamformer	FWHM (mm)
DAS	0.43/0.45
MV	0.18/0.28
SLSC	0.67/0.63
DA-MV SLSC	0.27/0.30

**Fig. 6:**
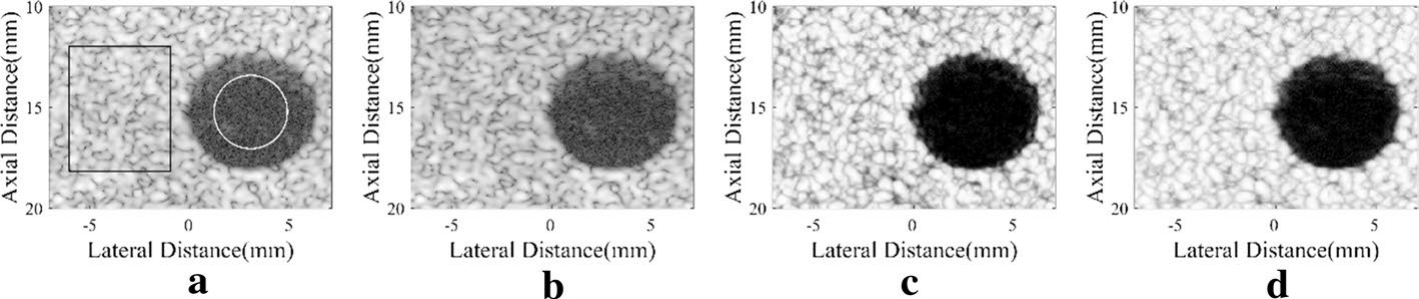
Experimental anechoic cyst: **a** DAS, **b** MV, **c** SLSC, **d** DA-MV SLSC. The **a** and **b** are B-mode images shown with a dynamic range of 60 dB

For the anechoic cyst phantom, results are shown in Fig. 6. The improvements of the proposed method mainly happen in less sidelobe noise inside cysts, clearer cyst margins and better speckle performance. Numerical results are presented in Table 5, which are calculated using the anechoic cyst marked by a white circle and its background speckles marked by a black rectangle. Similar to the anechoic cyst simulation, the MV shows no obvious differences in CR, CNR, and speckle SNR when compared with the DAS beamformer. With the SLSC method, the CNR is significantly increased, and the speckle SNR is also improved, which can be found in results of the SLSC and the DA-MV SLSC. In the comparison between two SLSC methods, the DA-MV SLSC obtains not only higher CR and CNR, but also better speckle SNR. This indicates that the proposed method is effective in suppressing background noises and enhancing the speckle performance.

**Table 5:** CR, CNR, and speckle SNR of the experimental cyst for different beamformers

Beamformer	CR (dB)	CNR	Speckle SNR
DAS	− 28.39	1.81	1.88
MV	− 28.23	1.84	1.92
SLSC	− 36.71	7.88	8.30
DA-MV SLSC	− 39.04	10.14	12.26

### In vivo study

**Fig. 7:**
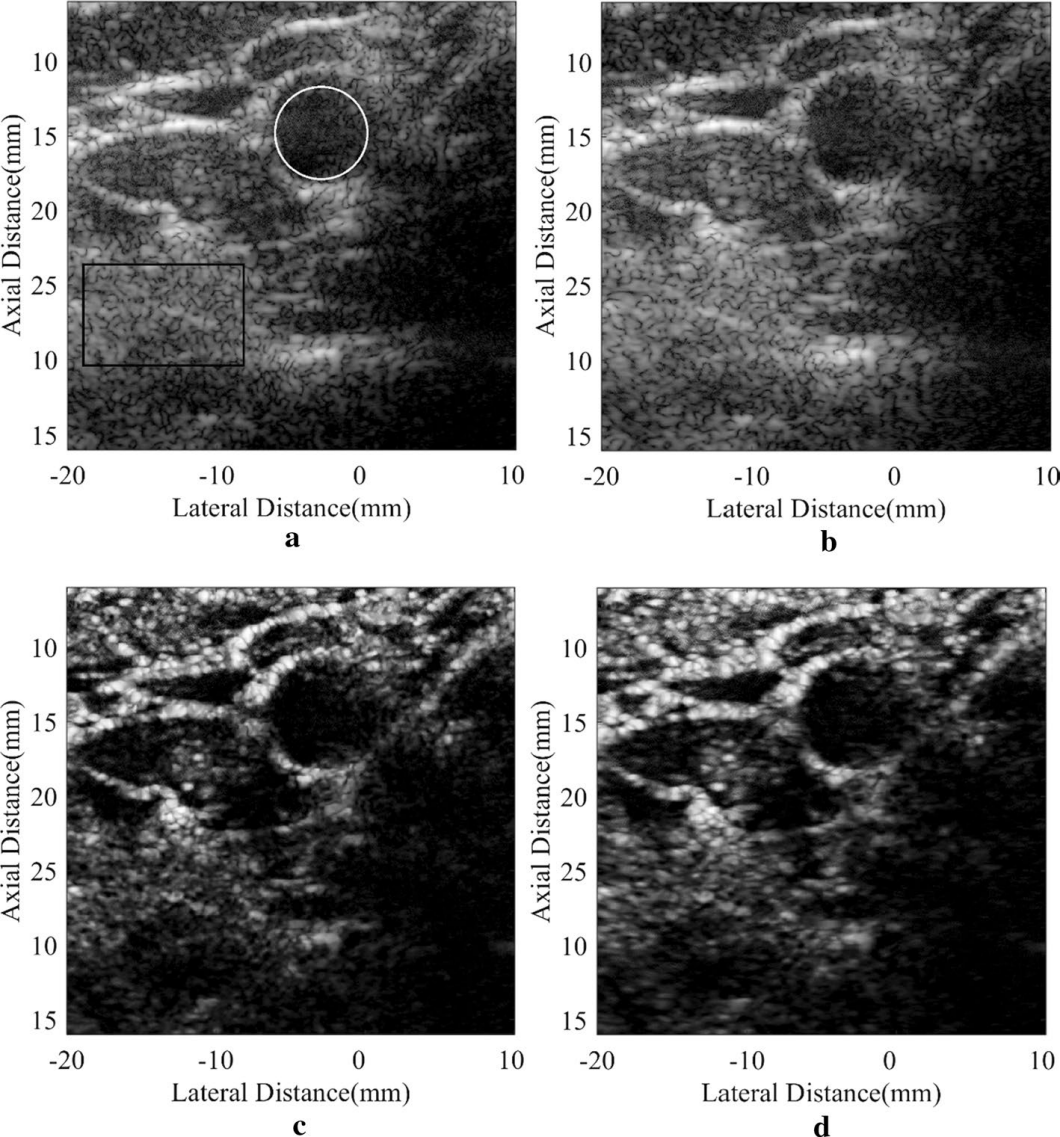
In vivo human carotid artery: **a** DAS, **b** MV, **c** SLSC, **d** DA-MV SLSC. The **a** and **b** are B-mode images shown with a dynamic range of 60 dB

In vivo images of a human carotid artery using different beamformers are presented in Fig. 7. The carotid artery has complicated anatomic structures, but the region inside the blood vessel has similar features with an anechoic cyst. Therefore, the same research process used in cyst simulations can be adopted to study the performance of these methods. As observed, noises inside the artery are obvious in B-mode images including the DAS one and the MV one. On the contrary, they are notably removed in the SLSC results. The artery wall can be better defined especially with the DA-MV SLSC. The comparison between the SLSC and the DA-MV SLSC demonstrates that the hyperechoic structures are more distinguishable with our method. The indistinct speckle regions in the original SLSC result are clearer with the proposed DA-MV SLSC, as well, which improves the visualization of the anatomical structures.

Again for the quantitative assessment, the inside-artery regions marked by a white circle and the background speckle marked by a black rectangle are adopted to calculate CRs, CNRs, and speckle SNRs. Corresponding results are given in Table 6. Compared with the original SLSC scheme, the proposed method brings an increased CR, along with a better CNR and speckle SNR.

**Table 6:** CR, CNR, and speckle SNR of the carotid artery for different beamformers

Beamformer	CR (dB)	CNR	Speckle SNR
DAS	− 15.20	1.27	1.56
MV	− 19.86	1.40	1.56
SLSC	− 20.14	1.46	1.70
DA-MV SLSC	− 22.05	1.78	2.01

## Discussion

The proposed method is an optimization of the original SLSC method. The utilization of the MV beamforming in both the Tx and Rx aperture is effective in enhancing the imaging quality. From the simulated and experimental results, the proposed method obtains a comparably better performance than other beamformers. The resolution and contrast improvements result from two aspects. First, the implementation of the adaptive beamformer (MV) brings a better synthesis for both apertures than the conventional averaged compounding, which could further improve the performance of the following SLSC algorithm. The inherent adaptivity of the MV method mainly contributes to the resolution of the output images [20]. In addition, the MV helps remove the off-axis noise around hyperechoic targets, which results in a decline of the spatial coherence in these regions. Since the SLSC process sums all short-lag spatial coherence, the noise part in the final image is also suppressed. Second, the spatial coherence is measured in not only the Rx aperture but also the Tx one, which makes the proposed method different from others. The DA-MV SLSC method is based on the acoustic reciprocity [16]. Thus, there is mutually dual between the beamforming processes in “Transmit aperture weighting for the SLSC imaging” and “Receive aperture weighting for the SLSC imaging” sections. The SLSC estimation in both apertures fully utilizes the coherence information in the synthetic aperture 2D echo data matrix. As a consequence, the contrast is further increased in comparison with the original SLSC scheme. In general, the high contrast along with a better resolution than conventional DAS and SLSC methods indicate that the proposed method could be a promising ultrasound imaging technique for better visualization of the anatomical structures and higher lesion detectability.

As for some parameters used in the proposed method, the subarray length *L* and the SLSC ratio *Q* are set to the same value for dual processes in order to accord with the acoustic reciprocity. Actually, using different values for the Tx and Rx aperture is also feasible since two processes in “Transmit aperture weighting for the SLSC imaging” and “Receive aperture weighting for the SLSC imaging” sections are mutually independent. Changing parameters for one aperture will not affect the beamforming process in another. It is a considerable issue that we can modify the final image by changing both apertures’ parameters separately. It might cause a degradation in the robustness but could bring advantages for specific situations. For instance, when the ROI contains several hyperechoic targets, enlarging *L* and decreasing *Q* for the Rx aperture can help achieve a higher contrast and better noise reduction around these targets, while the background speckle region could be damaged.

The computational complexity (CC) of the proposed method is a little higher than those of the MV beamformer and the original SLSC beamformer, since both the MV and the SLSC process are conducted in our method. The MV method has a complexity of ${\text{O}}\left( {L^{3} } \right)$ [25, 26], while the CC of the SLSC is comparably similar with that of the MV beamformer [2], also about $O\left( {L^{3} } \right)$. The proposed scheme includes two MV beamforming processes and two SLSC estimations, for dual apertures, respectively. Thus, the CC is about four times the original MV methods, but still at the level of $O\left( {L^{3} } \right)$. In consideration of the application of the graphics processing unit (GPU), it is possible to realize the real-time MV and SLSC algorithm [27–29]. We believe that our method also has the potential for real-time practical implementation.

There are still some unsolved issues in the proposed method. Above all, the integration of the MV and the SLSC could decrease the robustness in complicated situations. The hyperechoic cysts could be indistinguishable from the speckle region using the SLSC scheme. Another problem occurs in the clinical situation. The image enhancement of the proposed method could be influenced by tissue motions, channel noises, and phase aberration. Adopting a compensation technology for the tissue motion could be an effective alternative [30]. Future studies will focus on handling these drawbacks.

## Conclusion

We develop a novel SLSC imaging method adopting the MV adaptive synthesis on dual apertures in this paper. The synthetic aperture echo dataset is adaptively beamformed in the Tx and Rx apertures, and the spatial coherence is also estimated through both apertures. Simulated, phantom and in vivo data are collected to demonstrate the advantages of the proposed method. Results show that our method can enhance the image resolution which is usually damaged by the original SLSC algorithm. More than this, the CR and speckle SNR are also increased due to the utilization of the adaptive focusing process. Therefore, we consider that the proposed method could be a promising SLSC imaging technique for the better imaging quality in medical ultrasound imaging.

## Abbreviations

CC: computational complexity
CF: coherence factor
CNR: contrast-to-noise ratio
CR: contrast ratio
DAS: delay-and-sum
DA-MV SLSC: SLSC imaging using adaptive MV beamforming on dual apertures
FWHM: full-width at half-maximum
MV: minimum variance
PSF: point spread function
PWC: plane wave compounding
PWI: plane wave imaging
RF: radio frequency
ROI: region of interest
Rx: receive
SA: synthetic aperture
SLSC: short-lag spatial coherence
SNR: signal-to-noise ratio
Tx: transmit
